# Exploring the Religious Practice of Langar as a Route to Health Promotion in the Sikh Community in Northern England: A Qualitative Study

**DOI:** 10.1007/s10943-024-02077-6

**Published:** 2024-07-07

**Authors:** Amrit Daffu-O’Reilly, Kuldip Bharj, Maria Horne, Daryl O’Connor, Mark Conner, Felicity Astin

**Affiliations:** 1 School of Healthcare, University of Leeds, Baines Wing, Leeds, LS2 9JT England; 2 School of Psychology, University of Leeds, Leeds, LS2 9JT England; 3 School of Health, Wellbeing and Social Care, The Open University, Milton Keynes, PO Box 197, Buckinghamshire, MK7 6AA England

**Keywords:** Sikhs, Langar, Health promotion, Cardiovascular disease, Qualitative research

## Abstract

Cardiovascular disease and type 2 diabetes are prevalent among UK South Asians. Langar is a Sikh religious food practice that could be important in dietary health promotion. This study explored perceptions of langar, its role in health, readiness and strategies for change and whether Gurdwaras (Sikh place of worship) are able to support change. Using an exploratory qualitative design, we conducted eight focus groups and one single interview with 28 Gurdwara attendees (12 males, 16 females), analysing transcripts using thematic analysis. Four themes were developed that described the meaning of langar, the understanding of health among Sikhs, the evolution of langar and implications for health and changing langar to protect health. It can be concluded that any change to langar needs to be gradual, respectful and widely supported. This research has scope to continue in light of the potential impact on health for the Sikh community.

## Introduction

### Cardiovascular Disease and Type 2 Diabetes Among UK South Asians

The UK South Asian community experiences a 50% higher coronary heart disease mortality rate compared to White British people (British Heart Foundation, 2022) and is up to six times more likely to develop type 2 diabetes (Hanif et al., 2018). Cardiovascular diagnoses come at an earlier age (< 60 years) compared to White and Black patients (George et al., 2017). Sikhs form part of the wider UK South Asian community and share a similar risk profile. Improving the health of UK Sikhs, the wider South Asian community and people from other black and minority ethnic groups continues to be a public health priority evidenced by government health policies and the wealth of evidence and studies dedicated to this field both in the UK and globally (NHS England, 2023; Vafaei et al., 2023). The 2021 UK Census reported that 524,000 citizens identified as Sikh, an increase of 92,000 from 2011 (Office of National Statistics, 2021).

Risk factors for disease development among UK South Asians are well documented and complex, including sub-optimal diets, low physical activity (Sidhu et al., 2022) and ethnicity—as a socio-economic determinant of health inequality (Chouhan & Nazroo, 2020; Pursnani & Merchant, 2020). Health inequalities, however, differ among the South Asian groups (Chouhan & Nazroo, 2020). Further, public health campaigns often fail to impact seldom heard communities for a variety of reasons, for example, inadequate reach, contributing to additional health inequity (Illife et al., 2017).

Lifestyle change interventions to tackle conditions, such as type 2 diabetes, have achieved modest success in South Asian communities (Daffu-O’Reilly et al., 2017; Jenum et al., 2019). South Asians report universal barriers to lifestyle change such as time constraints, but psychosocial, cultural and religious influences also significantly shape health perceptions, responses to changes in health and health decision-making (Graham & Crown, 2014; Iqbal, 2023; Lucas et al., 2013; Sidhu et al., 2016). For example, cultural customs such as the offering and sharing of food occupy a unique position among South Asian groups and continue through generations (Fernandez et al., 2015; Sidhu et al., 2022). Fatalism, traditional beliefs, gender roles, family and community structure, that tend to be embedded within religion and culture, also encourage an approach to health that sits outside the scope of western health management (Mohamed et al., 2017; Oskopo & Riegel, 2021). Additionally, population-level health promotion may lack the specificity to effectively target minority ethnic communities’ perceptions of and influences on dietary intake and lack impact on changing ‘modifiable behaviours’ (Hipwell et al., 2015; Lucas et al., 2013).

### Langar in the Sikh Religion and as an Opportunity for Health Promotion

Langar—a sacred lacto-vegetarian, free of charge meal—in the Sikh tradition was initiated by the founder of the religion—Guru Nanak Dev Ji—to promote equality, oneness and charity. Sikhs, who originate mainly from Northern India, follow three religious principles: earning honestly (Kirat Karo), sharing generously (Vand Chakko) (including selfless service—seva) and remembering God (Naam Japo) (Kaur & Basra, 2022).

In the Gurdwara, a Sikh place of worship, the religious community (sadh sangat) meet for collective worship and to prepare and share langar, where preparing langar is a part of seva and eating langar is an expectation. All forms of seva have been associated with positive emotional and social well-being and promote a sense of community cohesion (Graham & Crown, 2014; Sohi et al., 2018). Langar is prepared in almost every Gurdwara around the world—there are over 300 Gurdwaras in the UK alone and many worldwide. According to the British Sikh Report (2022), 33% of UK Sikh women and 43% of men visit a Gurdwara at least once weekly (with 5% of people aged over 65 visiting daily).

Little is known about the nutrient composition of langar. It is prepared using the traditional food preparation methods as in the homes of Sikhs, though it is ‘richer’ than domestically prepared food. Oils, butter and ghee are used to prepare the main dishes and to fry foods and white sugar is used to prepare desserts and delicacies. This is relevant because it is well known that the South Asian diet is less than optimal (Emadian et al., 2017) and there is growing recognition among South Asians of the same (Tirodkar et al., 2011). Langar, as a shared meal with global relevance, presents as an opportunity for health promotion among Sikhs.

Typically, langar consists of a dry vegetable dish (sabzee), lentils (dhal), chapatti (unleavened bread), yoghurt and a dessert. ‘Pangat’ langars are everyday langars provided by a Gurdwara, the ingredients for which are supplied using the Gurdwara funds or donations. However, ‘sangat’ langars are requested and funded by a family from the sadh sangat to mark a special occasion. They are far more elaborate with a greater volume and variety of food served over several days including more delicacies, fried foods and desserts—this is the focus of this research and the potential impact on health. Langar is sacred—the first thali (sectioned stainless-steel plate) is offered to the Guru Granth Sahib Ji (holy scripture) and the ceremonial kirpan (short sword with curved, single-edged blade) passes through each item of food. The blessed food is returned to the remainder of food, thus blessing the entire food contents (Desjardin & Desjardin, 2009).

Previous, albeit limited, research (e.g. Coe & Boardman, 2008) investigating health in a Gurdwara setting indicates an interest in langar and health. Respect for langar may have deterred further research. However, this respectful investigation opens a new line of enquiry into health promotion for UK and global Sikhs and for the development of health interventions that are embedded in and sympathetic to religion.

As langar is prepared and shared in the Gurdwara, there is utility in exploring the Gurdwara as a health promotion venue. As well as contributing to mental and spiritual health (Agyekum & Newbold, 2016; Northridge et al., 2017; Tagai et al., 2018), faith-based interventions that draw upon faith principles and are implemented at faith settings can also improve physical health (Dunn et al., 2021; Kwon et al., 2017; Labun & Emblen, 2007; Lim et al., 2019; Litalien et al., 2022; Tristão-Parra et al., 2018). Places of worship, as settings for health promotion, have considerable potential because of their reach, familiarity and trusted leadership (Public Health England, 2016); however, these venues, especially Gurdwaras, are underutilised in the UK (Tomalin et al., 2019). There is a developing body of evidence that demonstrates the effectiveness of health interventions at faith organisations, especially for seldom heard communities and across different faiths (Burchenal et al., 2022; Lim et al., 2019; Juon et al., 2008; Schwingel & Gálvez, 2016; Whisenant et al., 2014).

### The Current Research

Since very little is known about langar and to be able to promote the health of Sikhs, it is first necessary to understand how langar is understood to identify any scope for change. Therefore, in this novel research, our aims were to explore the perceptions of langar as a religious ritual, its role in health, readiness for a new way of preparing and sharing langar, strategies for change as well as the suitability of the Gurdwara to support this.

## Methodology

### Design and Sample

An exploratory descriptive qualitative approach was appropriate to investigate participants’ experiences and views because little is known about this topic (Swedberg, 2020). The transcripts were analysed using the data-driven approach of inductive thematic analysis (Braun & Clarke, 2022) given the lack of relevant theories and evidence base for this topic, permitting both descriptive and interpretive accounts of the data.

The consolidated criteria for reporting qualitative research (COREQ) checklist items were used (Tong et al., 2007).

The COVID-19 pandemic lockdown and the lack of experience with research among Sikhs presented challenges to recruitment. We aimed to recruit a purposive sample of participants, balanced by gender, from two Gurdwaras located in the North of England. We partnered with two Gurdwaras (A and B) resulting from existing relationships, similarity in size, willingness to be involved and geographical location. The research was advertised more widely to include people who did not have a primary affiliation with the partner Gurdwaras (C) through personal contacts and social media.

The principal contact at each Gurdwara facilitated purposive sampling of potential participants through social media, word-of-mouth, individual approaches and the distribution of study information. Interested participants consented to their contact details being shared with the researcher who then contacted them to provide study information and undertake screening (eligibility was all Sikh adults, over 18 years of age who attended a Gurdwara in the target geographical area). Participants were sent written information and a consent form (verbally explained in Punjabi if necessary). In total, eight focus groups and one interview were conducted with 28 individuals between November and December 2020. One South Asian, female researcher completed all focus groups and interviews. It is estimated that data saturation can be achieved after six interviews (Guest et al., 2006).

### Data Collection

The COVID-19 lockdown prohibited face-to-face data collection; videoconferencing was chosen as the next best alternative to face-to-face interviews, providing an option for telephone calls for those who preferred this method considering social restrictions. Challenges around low digital literacy, connectivity (Carter, 2021) and potential apprehension because of topic sensitivity were anticipated. The interviewer emphasised that there were no ‘right’ or ‘wrong’ answers and ensured that all participants were given the opportunity to contribute. The data collection environment afforded a safe virtual space for these first-time discussions to take place.

The interview schedule was developed to reflect the research aims (Roulston, 2010) given the novelty of the subject. An English version was piloted with two English/Punjabi speakers, translated by three researchers fluent in Punjabi and then piloted again with three different Punjabi speakers. Revisions were made at each iteration. Participants chose to join in a focus group or individual interview conducted in the language of their choice (English, Punjabi or combination). To minimise digital exclusion, coaching on videoconferencing was provided.

Informed consent was obtained from all participants included in the research and demographic data were collected after consent was obtained. Focus groups and interviews were audio-recorded using the online platform recording facility or a digital recorder for telephone interviews. Participants using the online platform chose to have their cameras ‘on’. Audio files were downloaded and saved securely on the University OneDrive before being sent for professional translation and transcription by bilingual transcribers. Sample translations were assessed for quality and accuracy by the researcher conducting data collection before all the transcripts were generated (Swartz, 2014).

### Data Analysis

Using Braun and Clarke’s (2022) method for thematic analysis, familiarisation with the dataset was achieved by reading and re-reading transcripts. Individual codes were generated from each transcript, leading to the development of initial candidate themes and sub-themes. Themes and sub-themes were reviewed to ensure that they reflected coded extracts and lastly, themes were finalised and named. NVIVO (version 12) was used to support the management and retrieval of the data.

To ensure trustworthiness, four researchers, experienced in qualitative research, independently coded four transcripts and met virtually to compare codes as a way of exploring multiple interpretations of the data (Morse, 2015). As part of the reflexive process, the research team (South Asian and White) discussed how their own identities, social positions, religious understandings and experiences could influence their interpretations, for example, views about fatalism and control over chronic disease processes (Evans et al., 2017). Instances in which research participants’ views differed from the majority were discussed to support the robustness of the data analysis (Thomas, 2006). The two Sikh researchers were able to provide contextual understanding of the data and describe the meaning of certain phrases because of familiarity with the language.

### Ethical Considerations

This research was performed in line with the principles of the Declaration of Helsinki. Approval was granted by the Ethics Committee of the University of Leeds (September 2020, Grant Number TR2432).

Participants were briefed about confidentiality, data protection and withdrawal from the research. Informed verbal consent was recorded. Participants were requested to respect the confidentiality of others. During interviews, the researcher observed for signs of participant distress and invited participants to make contact if they had concerns.

## Results

A total of 44 participants expressed an interest in participating in the research; 32 consented and of these four withdrew because of ill health. The final sample comprised of 28 participants, (See Table 1 for participant demographic characteristics) who contributed to either a focus group interview (eight focus groups with 2–5 participants in each) and one individual interview. A total of four focus groups were conducted in Punjabi and four using a mix of English and Punjabi. The single interview was mainly in English. The focus groups and interview duration averaged 68 min (range 60 min to 76 min that included briefing, verbal consent, interview and debrief). No participants became distressed or noted concerns during the research process.

### Participant Demographic Characteristics

Table 1 presents the demographic characteristics of the sample.

**Table 1:** Participant demographic characteristics

Characteristic	*N* (%)
*Participants*	
Males Females	12 (43) 16 (57)
*Age*	
40–49 50–59 60–69 70–79	4 (14) 2 (7) 12 (43) 10 (36)
*Gurdwara membership*	
Gurdwara A (four focus groups) Gurdwara B (three focus groups, one single interview) Other Gurdwaras (one focus group)	14 (50) 12 (43) 2 (7)
*Occupation*	
Information Technology Clinical/medical Administration Managerial Business owner Driving instructor Civil servant Retired Activities co-ordinator	1 (4) 1 (4) 1 (4) 4 (14) 2 (7) 2 (7) 1 (4) 15 (52) 1 (4)
*Gurdwara Attendance*	
≥ 1 time a week Once a month Twice a month Every 2 months Few times a year	22 (78) 1 (4) 2 (7) 1 (4) 2 (7)
*Langar consumption*	
On every visit At least once a week Most times when visiting Once every couple of weeks Never	17 (60) 5 (18) 3 (11) 2 (7) 1 (4)
*Long-term conditions*	
None Type 2 diabetes Hypertension Kidney problems Fibromyalgia Pre-diabetes Hypothyroidism Arthritis Blood disorder Sleep apnoea Asthma Cholesterol	14 (38) 7 (18) 7 (18) 1 (3) 1 (3) 1 (3) 1 (3) 2 (5) 1 (3) 1 (3) 1 (3) 1 (3)
*Country of birth*	
UK Kenya India	5 (18) 9 (32) 14 (50)
*Duration in UK*	
All of life ≥ 20 years ≥ 40 years ≥ 50 years ≥ 60 years	5(17) 1 (4) 7 (25) 12 (43) 3 (11)

### Themes

**Fig. 1:**
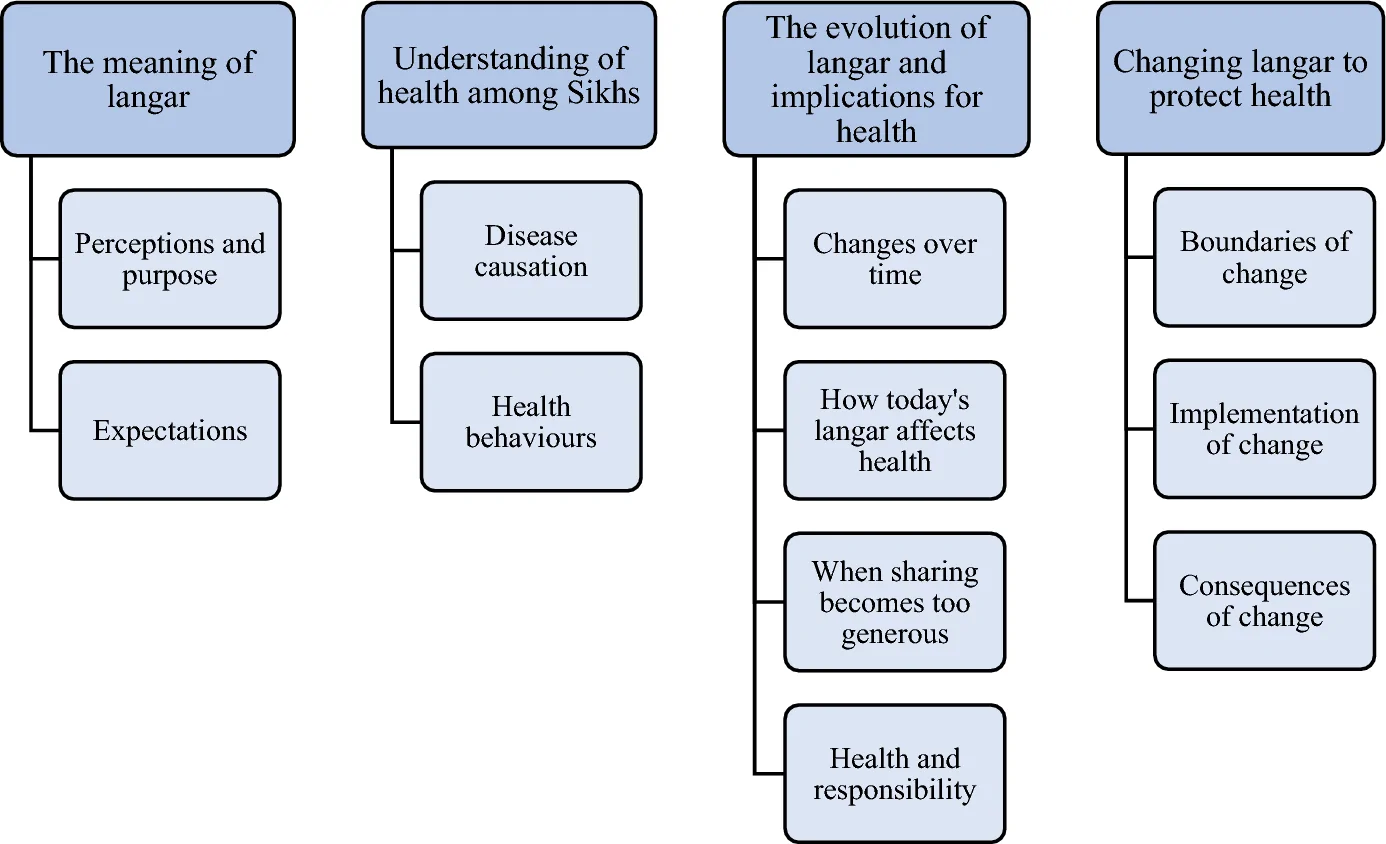
Thematic map of themes and sub-themes

Four themes were derived from the data analysis, with supporting sub-themes described below. Figure 1 presents a thematic map of the findings.

#### The Meaning of Langar

##### Perceptions and Purpose

Since perceptions of langar are un-evidenced, we provide useful and brief context as to how langar is perceived.

Langar was consistently discussed with great reverence and respect, with emphasis placed on its uniqueness among world religions. Visiting the Gurdwara, eating langar and recalling the taste all form a part of childhood memories. Most older (> 65 years) participants recalled the events leading to the inception of langar by Guru Nanak Dev Ji—the Sacha Sauda (the true bargain), though younger participants were unfamiliar with the Sacha Sauda. Irrespective of knowledge relating to the origins of langar, there was strong affirmation that langar started as a humble meal to feed the needy and is a tradition that is practised to this day. The concept of humbleness was emphasised as an important feature of langar as well as the concept of ‘giving’ to benefit others and the less fortunate, linked with the tenet of ‘Vand Chakko’.

When Guru Nanak Dev Ji went for Sacha Sauda…when his father, had given him 20 Rupees...he had met some holy men who were hungry. So, what he did was, he brought some three or four things…ghee, sugar, flour, fire and water...these were the five things he gathered. He made food from these and fed them…so, this tradition carried on in the Gurus’ homes [Gurdwaras]. (Female 3, Gurdwara A, aged 74)

However, one participant felt that langar should only be prepared for, and consumed by, Sikhs, except in the case of charity because it is sacred and needs to be respected in accordance with the Sikh faith.

The preparation, serving of langar and maintenance of the langar kitchen forms another important part of the Sikh religion—seva—selfless service. There are many forms of seva in addition to preparing langar; providing the ingredients, serving, washing dishes, cleaning and tidying all contribute as well as other activities—therefore being involved in langar provides an opportunity for seva. Seva is a stand-out feature of the Sikh religion.

So, yeah, I totally agree with everybody else that has said anything. I think the most important part of langar was initially…seva and seva is the biggest thing in Sikhism. (Male 1, Gurdwara A, aged 61)

The consumption of langar when visiting the Gurdwara breaks down social status and hierarchy, creating equality and oneness because everyone eats together, either sitting on the floor or at tables where no regulation or guidelines on seating exists. The mandatory consumption of langar when visiting a Gurdwara started with the third Guru, Guru Amar Das Ji, when even nobility was required to sit with lay people and eat langar prior to meeting with the Guru.

For me the meaning of langar is that when we eat langar in the Gurdwara, we are equals. All of us eat together as equals, regardless of one being poor or rich. When Emperor Akbar went to see Guru Amar Das Ji, even he ate langar sitting with the poor, as an equal. So, langar makes us equals. We sit and eat together. (Female 16, Gurdwara B, aged 63)

##### Expectations

The requirement to consume langar by Guru Amar Das Ji still stands today. With langar representing a holy offering, custom dictates that langar should be gratefully received, respected, not criticised, refused, questioned or ‘assessed for quality’. Refusing langar could be considered disrespectful and breaking with tradition, therefore at least a small amount should be consumed. In addition to the expectation to eat langar on every visit, there is an added expectation not to waste it because it is sacred, perhaps indicating the ease at which overeating can occur. Leaving food is considered immoral and even a ‘paap’ (sin).

...and you have that conditioning that you don’t waste Guru ka langar. You don’t let it go to waste; you end up consuming it most of the time. (Male 4, Gurdwara C, aged 65)

Langar, therefore, is an important part of the Sikh tradition and identity, symbolising the concept of equality and community. The practice of langar promotes selfless generosity, but the customs around consumption are perhaps not optimised to healthy intake, especially in relation to portion size. However, the data in Table 1 shows that not everybody ate langar on every visit to the Gurdwara, despite the general expectation to do so.

#### Understanding of Health Among Sikhs

##### Disease and Causation

Participants identified with ease the long-term conditions that affect the Sikh community—heart disease, diabetes, hypertension, mental health problems (especially depression) and alcohol addiction—were named. Modifiable risk factors such as the Asian diet, lifestyle, stress, overweight and obesity as well as non-modifiable risk factors such as old age, increasing frailty or ‘weakness’ associated with ageing and cold climate were mentioned.

For some, advancing age lent itself to the ‘inevitability’ of acquiring disease because of the perceived association between ageing and weakness/frailty, but unhealthy lifestyle habits could further exacerbate poor health. Diseases sometimes just happen with advancing age, with people responding by managing their conditions, or not. There was sense that acquired diseases cling to the individual, with little or no chance of reversal. This in turn may encourage disengagement from self-management.

Diseases can happen on their own because of old age too, right? Like diabetes…when you are young you eat anything, you exercise, move about and work...and when you cross 50, all you do is eat and sit, eat and sit. Diabetes happens many a times on its own after 50 or 60. Some people control it by diet. Some people just go on eating. So naturally, the body keeps on getting weaker. So, because of age you tend to get the diseases then. And you don't get rid of them then. (Female 3, Gurdwara A, aged 74)

##### Health Behaviours

Younger participants (referring to older people) felt that the Sikh community tended to ‘wait’ for diseases to appear and individuals would be surprised when diagnosed with an illness. Some participants reported modifying cooking techniques at home, due to ill health or to be proactive, by restricting intake of fried foods, switching to healthier oils and incorporating ‘whole wheat’ chapatti flour into their diets. Nevertheless, old habits prevailed when guests or family members visited, where the custom of serving rich foods takes precedence and portion sizes are not important, and ‘filling’ the guest is. It was felt that ‘Indian thinking’, referring to feeding and nurturing, applies when hosting others and presents as a genuine barrier to change:

…now that’s the mentality. I remember when my wife’s grandma, when she was alive, it was just constantly feeding and feeding…until like you can’t eat anymore…and you’ve still got stuff in your mouth…and you’re still being fed. Please just stop! Leave me alone!

…that’s an Indian thing, isn’t it? (Male 2, Gurdwara A, aged 44 and Female 2, Gurdwara A, aged 57)

This concept of feeding is a demonstration of love, affection and generosity, but is also deeply anchored to traditional perceptions of health, linking increased body weight with status and wealth—for many older or more traditional Sikhs, this perception continues to shape thinking and eating behaviours. Younger people tend to reject/challenge this type of thinking and behaviour, but change seldom occurs because of respect for elders and to preserve hierarchical boundaries.

### The Evolution of Langar and Implications for Health

#### Changes Over Time

All participants, except one, noted that langar has changed over time, from a humble and simple meal to an elaborate one, especially in western countries, thanks to increased economic wealth, human effort and resources. Sangat langars have become ‘fancy’ with increased volume, variety and opportunities to eat, e.g. breakfast, surpassing the variety available in restaurants and leaving little room or time for digestion.

Now, we go to the Gurdwara...there are about five to six varieties [of snacks] in the morning, with tea. You'll find pakoras, samosas, balu shahis [deep fried doughnut], gulab jamuns [sweet fried dumplings], jalebis [deep fried sweet snack]...two to three types of sauces...tamarind sauce, tomato sauce, and chilli sauce...this makes it seven varieties. And then in the afternoon in langar – fulka [chapatti], dhal, sabzee, bhallas [deep fried fritter]...there're so many things. You don't get that much even in a restaurant. (Male 9, Gurdwara B, aged 74)

Even in the absence of a sangat langar, tempting food items are available in the Gurdwara and managing this is a challenge:

When we visit a doctor, the doctor tells us to not eat certain things...to avoid getting this illness, avoid eating these things. Whereas here...no one avoids eating anything. In our Gurdwara, there are three to four types of pickles. A patient like me, maybe he won't eat at home, but will relish eating there. Why? Because one cannot resist. (Male 6, Gurdwara A, aged 70)

During the 1960s and early 1970s, langar was seldom served because functioning kitchens were not available; instead, a small portion of nuts/dried fruit was provided as a blessing. For those who could recall this earlier time, the change in langar was considered significant with indications of disapproval with the langar of today.

#### How Today’s Langar Affects Health

On account of the ‘heaviness’ (dense ingredients) of langar (because of oil, butter, ghee, sugar and frying) and amount of food available (and consumed), there were concerns about the effects of langar on health. There is often visible surplus oil on food that needs to be scooped out and langar was described as difficult to digest by many, in addition to causing lethargy.

The langar is very heavy. Ghee, milk and then the tadka [base sauce]. Many a times you see the oil floating on top. And many a times one has to scoop out the oil. Sisters scoop out the oil like this. Then it's all very heavy, and it's not healthy. (Female 3, Gurdwara A, aged 74).

Participants were clear about the dangers of high oil/butter/fat consumption, especially among those who had existing conditions, but all these foods are readily prepared and consumed at the Gurdwara, mainly out of habit and expectation.

As [brother] has said, it's [langar] not good for health...diabetes patients...I myself am a diabetic, right? Some other people have many other problems...problems of heart...other health issues...the fried foods like samosas and pakoras, right? Sometimes there are muthhies [flat, fried savoury biscuits], sometimes salted seerni [fried savoury bites]. All these things have oil. Extreme amounts of oil are used. Similarly, they throw blocks of butter in sabzees. (Male 5, Gurdwara A, aged 70)

Achieving healthy eating in the context of religious and traditional customs was described as difficult. Common ingredients—ghee, oil, butter, sugar and spices—were all identified as potentially harmful foods. When preparing saag (spinach), copious amounts of butter are used to achieve the required taste, the spinach is over-cooked and more butter is added on top of the saag as it is served as well as on fulkas. One participant reported enjoying eating saag, but consequently reflected upon his meal having seen the numerous discarded empty boxes of butter outside the langar kitchen.

Whilst problematic ingredients in langar are recognised, a dilemma becomes apparent because most of them are *needed* to make langar. The use of white sugar is practically unavoidable for sweet dishes and healthier alternatives have never been considered or might not be feasible.

But then the desserts we make, how can we make them healthy? Sweets will have all that needs to be added in them. There will be as much sugar too. As much butter too. We can't make it very healthy. (Female 9, Gurdwara A, aged 73).

Today’s langar encourages overeating because of the quantity of food available coupled with the relaxation of self-control on special occasions. Health is put on hold until festivities are over and can be ‘restored’ later or managed with extra medication or promise of exercise. This typically means temporarily ‘forgetting’ about normal restrictions that may apply in relation to dietary intake.

…If we think health wise, if we think about our health...people do eat langar there...they get two servings of kheer [rice pudding] too, but if they have diabetes, they'd say - I'll take another pill at night. Plus, I’ve got some ginger. I'll eat ginger, so that my sugar levels come down. (Female 4, Gurdwara A, aged 60)

Some participants linked the frequency of langar consumption to health, suggesting that those who spend more time at the Gurdwara are at greater risk of diet-related poor health and a once-a-week rate of consumption was not unhealthy (and therefore change is not necessary).

…the fact that I may eat langar four times a month if I go to the Gurdwara every week um, that’s fine. Langar is perfectly healthy. If you are a granthi [ceremonial reader of the holy scripture] or somebody who spends all their time in the Gurdwara, and you’re going every day and you’re eating that food every day, I would say it’s unhealthy. (Male 3, Gurdwara C, aged 41)

A further recent development in langar also caused concern for the health of children and young people. Pizza and other convenience foods are routinely provided to children attending educational classes at the Gurdwara. Greater effort should be made to provide healthy food for children and not be attracted by convenience.

Therefore, encouragingly, nearly all participants identified how langar could affect health, though one participant felt that it could not harm health because it is sacred.

#### When Sharing Becomes Too Generous

The difficulty with establishing the line between ‘generous sharing’ and ‘showing-off’ was apparent. Whilst sharing is an important part of the Sikh religion, it is also intimately linked with a demonstration of social status and a financial ability to be generous. People want to be perceived as generous, but when does generosity blur into competition and a need to demonstrate social status? Langars are expected to at least match, or outdo the previous langar resulting in more extravagance, competition, cost, wastage (despite the customs about wastage) and pressure to conform.

It [langar] should be simple as possible, but we do, I think people go overboard with it, they really do; and it’s all about being charitable as well but I don’t think that signifies being charitable. (Female 14, Gurdwara B, aged 60)

Participants felt that this type of thinking needs to be challenged and addressing this will be an important component of long-term change. A shift in mindset will support more responsible and healthier decision-making.

#### Health and Responsibility

Participants explained that under no circumstances can langar be stopped because of its reverent and important role in the Sikh faith. Then, discussions turned towards who should be responsible for health.

Concern for the future health of children/young people meant that the current generation need to change how langar is perceived and consumed to foster new habits and practice. There was a strong feeling of responsibility, using the collective ‘us’ rather than placing responsibility on any one person or other group of people.

...but, you know it is, it has to be on us, and it has to be how we have to change our mentality to be able to change the mentality of the next generation. (Female 2, Gurdwara A, aged 57)

Participants recognised that health was a personal responsibility characterised by making healthy lifestyle choices and exercising restraint. Simultaneously, the Gurdwara representatives felt that the sangat’s health was also the responsibility of the Gurdwara and sevadars (volunteers) by ensuring the provision of healthier food in sensible amounts:

...The jathedar [person in charge of kitchen/langar] in the kitchen should get some sort of training about how to make it healthy. And it's the responsibility of the individual as well. I think it should be the responsibility of both...if there are two to four items in the langar, you can eat fulka with one item, you don't have to take everything. You should be conscious of your health. So, on one side it's the responsibility of the individual, and on the other, the jathedar in the kitchen has the responsibility of cooking everything healthy. (Female 16, Gurdwara B, aged 63)

Almost all the participants believed that some reform to langar was needed to protect health and that health is the responsibility of everyone involved in langar—producers and consumers.

### Changing Langar to Protect Health

#### Boundaries of Change

Participants wondered if it was possible to change langar, given the number of ‘necessary’ essential ingredients. The experience and taste of langar was pertinent; enjoyment or taste of the food should not be compromised. The taste, however, is the result of traditional cooking techniques, leaving little room for modification. A very small minority voiced that a change to the taste of langar would be welcomed to protect health.

In recognising the barriers to change, especially in the context of taste, the most popular suggestion to make langar healthier and realign it with the original principals of Guru Nanak Dev Ji, was to return to a ‘simple’ and consistent langar, resulting in less langar production. To achieve this, the sangat need to be reminded to think about why they visit the Gurdwara.

I think this is where we’ve lost the whole essence of the langar, the langar in the Gurdwara should be consistent no matter what because it’s the langar, you know from the Gurus, you know why do you go to the Gurdwara? (Female 2, Gurdwara A, aged 57)

Additional benefits of a simple langar were identified—reduced consumption, time, effort, energy usage and the creation of more time for prayer. Some participants were keen, in the spirit of ‘sharing generously’, to donate the monetary difference between a simple and elaborate langar to charity.

To reduce variety, a practical set-menu system was recommended for *all* langars (pangat and sangat), although some felt that a few extra treats should be offered on very special occasions, such as the Gurus’ birthdays. Cost savings resulting from reduced langar could be invested in higher-quality ingredients, especially healthier oils and fresh produce. Fried foods traditionally offered for breakfast should be substituted with cereals. ‘Roving serving’—when more langar is offered by sevadars to those already eating—should be reduced and financial deterrents potentially introduced, e.g. for extra frying.

#### Implementation of Change

Some participants supported strict enforcement of change by the Gurdwara whilst others favoured a gentle, gradual and inclusive approach. Eventually, most participants leaned towards a more consultative approach to garner support, inclusivity, democracy and sustainability, whilst minimising upset.

Expectations and thinking around langar need to be ‘reset’—educational/lifestyle support should be offered to support and encourage healthier choices for sangat langars:

...hold classes in the Gurdwaras...classes for sangat too should be held the same way, where...sangat needs to be educated through…let’s say the television or projector. Because they are the ones who are going to prepare and hold langars. When they themselves will get educated on the subject...they will be somewhat careful if we educate them about langar. So, this is a very good thing. (Male 11, Gurdwara B, aged 64)

In contrast to the enthusiasm for change, there was hesitancy from the Gurdwaras to lead any change, preferring to adopt a supportive position. Changes to langar may bring disruption and aggravation to local/Gurdwara politics and reputations; therefore, an impartial third party would need to lead any change.

#### Consequences of Change

Participants agreed that a commitment to changing langar could bring opportunities for cohesiveness among Gurdwaras, supporting health across regions and nationally. Equally, change could also make the Gurdwara unpopular; the sangat could go elsewhere if requests were not fulfilled, impacting the size of the congregation and Gurdwara income. The potential negative consequences of implementing an ideal langar were expressed:

…dhal, kata [yoghurt] or a sabzee with um, one roti [fulka]…but I, I don’t think it’d be er, accepted very well by the sangat and I wouldn’t have much sangat actually [chuckles] if I did that! (Male 10, Gurdwara B, aged 75)

The only way to facilitate change and protect congregation numbers and income was for all Gurdwaras in a locality to participate in the change, though acknowledged that this may be difficult. Previous attempts to change langar have been unsuccessful—health promotion initiatives led by national and community organisations had short-lived effects due to difficulties in breaking habits, changing traditions, disappointment from the sangat and managing expectations, resulting in eventual relapse to ‘normal’ behaviours.

Organisations have visited our Gurdwara at times. The diabetes people [nurse practitioners] have also come… they have also approached many a times and have said that you should prepare healthy food…we pay heed for some time, and then it goes back to the same thing. (Female 7, Gurdwara A, aged 67)

Change could be met with resistance and difficult to maintain. However, exemplars of good practice were highlighted when discussing how other Gurdwaras have enforced changed, albeit with initial resistance and eventual acceptance. Long-term commitment and investment by researchers with the Gurdwara and the sangat would be essential to ensure new practices are accepted and embedded.

## Discussion

In this research, we have presented findings about the perceptions of langar, its role in health, willingness for change as well as practical solutions on how to make langar healthier to protect the health of the community. We have demonstrated that whilst the Gurdwara cannot ‘lead’ change, it is a promising setting to promote health, but there are boundaries of change that must be respectful to and sympathetic of religion and custom. Crucially, we found that the religious significance of langar is not a barrier to its modification, complementing research completed by Chapman and colleagues (2011) where Sikhs were able to identify the ‘western’ nutritional components as well as traditional elements of their food. Nevertheless, change could be challenging and a leadership team will need to be established.

Implementation of any change needs to be adopted by all Gurdwaras in a locality, at least, to have a unified and fair approach that will benefit all Gurdwaras and the sangat in a particular area. These new findings suggest that a new way of working with langar may be possible and that Sikhs are keen to make healthier choices when preparing and sharing langar, though traditions, expectations and customs could be barriers to change. This research presents findings that contribute to informing typologies and frameworks that are currently used to develop and adapt interventions so that they are culturally and religiously appropriate (Kwon et al., 2017; Liu et al., 2012).

This research makes a significant contribution to the sparse literature on langar as a route to health promotion by presenting a clear explanation of why langar, and eating behaviours more generally, might be difficult to change. Future work involving deductive analysis/mapping of transcripts to current behaviour change frameworks and models such as the Theoretical Domains Framework (Atkins et al., 2017) and COM-B model (Michie et al., 2011) will enable the development of theoretically informed interventions, though the capacity of the frameworks and models to accommodate cultural and religious specificity will need to be ascertained.

The cultural factors that influence health among South Asians should not be underestimated—a recent review by Iqbal (2023), exploring the influence of culture on eating behaviours in the context of type 2 diabetes across 29 studies, reported that food norms play a significant role in dietary decision-making. This places great importance on understanding the cultural and religious influences on food choice in developing interventions that are meaningful and likely to have more impact. It will be necessary to address long-standing traditions relating to sharing food, where the need to feed supersedes hunger and hosts need to satisfy a requirement to feed guests even if they are not hungry.

This research also makes strides in showcasing the Gurdwara as a promoter of community health. Other health research focussed on Sikh populations utilise Gurdwaras as collaborative community partners and locations for interventions for Sikh people (Islam et al., 2014; Northridge et al., 2017); our research places the spotlight on Gurdwaras as ‘agents’ of change to protect community health and to co-design interventions that ‘make sense’.

The literature shows that faith-based or placed health promotion activities are particularly effective in the prevention and or management of diseases where lifestyle modification plays a key role (McManus, 2017). Simultaneously, they address health inequalities by reaching those who may be less inclined to engage with mainstream health services because of known barriers, such as language and lack of religious or cultural context. After all, langar is not an ‘everyday’ eating behaviour, it is underpinned by religion.

There is just one published study about langar to compare and contrast the current research. Coe and Boardman (2008) report a week-long healthy lifestyle initiative ‘Apnee Sehat’ (Our Health) at a Gurdwara, including the results of four in-depth interviews. Similar to the current research, the Gurdwara was considered a suitable place to promote health. Changes to langar, however, were not well described. Apnee Sehat was also a short-term intervention without theoretical underpinnings and no investigation into the potential barriers and enablers to changing langar; a gap that the current research has successfully and comprehensively addressed.

There is clear utility in trying to promote health through religion—affiliation with a faith deters engagement with risky and harmful behaviours and those who practise faith tend to have an increased network of social support—all of which are correlated with positive physical and psychological outcomes (Litalien et al., 2022). Faith-based health promotion can have an important and impactful influence on health behaviours, since understandings and perceptions of health, as well as health behaviours, are intimately connected and rooted in faith and culture (McManus, 2017). Conventions pertaining to maintaining good health and addressing poor health are also firmly anchored to culture and faith.

Insightful evidence by Labun and Emblen (2007) highlights the intimate link between the Sikh religion and decision-making in relation to health, such that faith and spirituality guides daily activities and routines in the pursuit of living a healthy life and abstaining from unhealthy behaviours. Waking early, bathing and setting aside time for scripture and spiritual contemplation benefit both physical and emotional health and discipline the individual to make those healthful choices every day. Abstaining from eating meat, alcohol and avoiding other ‘destructive’ health behaviours is also informed by faith. In addition to having faith in ‘God’ and ‘His will’, there is also universal acceptance that illness can be caused by one’s own actions and it is individual responsibility to take any necessary action to protect health. Therefore, in recognising that food plays an important role in the context of religion and the pursuit of a healthy life, modifying langar could be transformative in the health of the Sikh population. Reframing langar within the context of faith, spirituality and health may be an effective strategy to support the creation and consumption of healthier langar, knowing that seva already plays an important role in mental and emotional health (Sohi et al., 2018).

In modifying langar, there is scope to promote healthy eating within the home too. The concept of commensality, hospitality and sharing food extends to the homes of Sikh people and other South Asian groups. Rich foods are often served to guests to indicate status, to welcome guests and similarly to langar, there is an expectation to eat what is presented and refusal can seem indecorous (Iqbal, 2023). Encouraging the sangat to think about langar differently, through the lens of health and religion, might be a catalyst to change at home too and how food is perceived and shared. Traditions are difficult to change; community/societal level endorsement would be required for maximum impact. Encouraging Sikhs to at least think about it is a good start.

Challenges to changing langar are expected, but there is also confidence that change can be achieved. The final outcome may not result in the nutritional optimisation of langar, but may make significant strides in producing a *healthier* version that preserves taste, encourages healthier choices and, crucially, respects religion, tradition and identity.

## Implications

This research raises implications for policy, research and practice. New light has been shed on the perceptions of a holy offering and contributes to a deeper understanding of why changing dietary habits is difficult for this, and other South Asian communities. Dietary guidelines need to be sympathetic of the symbolic, cultural and religious significance of food. Implementation of change will require investment and impartial leadership and opportunities for further research are plenty, timely and warranted. This research is informative for researchers developing langar-based healthy eating interventions at Gurdwaras.

## Limitations

The limitations to this research are the sample size. Nevertheless, this was an exploratory study and findings were ‘sense-checked’ with a sub-group of participants once restrictions were lifted, confirming concurrence with our results, though more consensus work is needed.

## Reflexive Considerations

The principal author, ADO, acknowledges that her social position as a Sikh, a researcher and someone personally invested in the health of Sikhs may have influenced data collection and interpretation. ADO is aware of the customs and symbolism relating to food, introducing what could be considered potential bias into the research process. The authors, however, consider this insight to be beneficial to providing cultural and religious contextual understanding of the data, leading to accurate and sensitive interpretation (Swartz, 2014). Nevertheless, to ensure robust analysis, findings were thoroughly discussed with all team members that consisted of Sikh and non-Sikh researchers. Finally, offering participants the opportunity to speak in Punjabi not only improved access to the research, but also afforded full expression and interpretation that may have not been possible in English for some.

## Conclusion

Little was known about langar; even less was known about the potential of promoting health through this religious practice. This research provides understanding about an important religious food practice for Sikhs and how it may be a route to health promotion at the Gurdwara. Exploring commensality provides useful insight into perceptions of food and food practices which is important for policy developers. This research is an exemplar of how health inequalities in research participation can be addressed through inclusion, engagement and access and that reaching seldom heard communities through faith to promote health is both successful and justified (Newlin et al., 2012).
